# Computational Methods Summarizing Mutational Patterns in Cancer: Promise and Limitations for Clinical Applications

**DOI:** 10.3390/cancers15071958

**Published:** 2023-03-24

**Authors:** Andrew Patterson, Abdurrahman Elbasir, Bin Tian, Noam Auslander

**Affiliations:** 1 Genomics and Computational Biology Graduate Group, Perelman School of Medicine, University of Pennsylvania, Philadelphia, PA 19104, USA; 2 The Wistar Institute, Philadelphia, PA 19104, USA; 3 Department of Cancer Biology, University of Pennsylvania, Philadelphia, PA 19104, USA

**Keywords:** cancer genomics, mutation signatures, machine learning, bioinformatics, clinical predictors, cancer drivers

## Abstract

**Simple Summary:**

Cancer is a complex disease that develops over time through accumulated mutations in DNA that transform normal cells into a cancerous state. To fully capture the complexity of the cancer genome, computational methods have been developed to summarize the mutational patterns of cancer, distinguish causal oncogenic mutations, and determine clinically useful mutational patterns. In this review, we survey different computational approaches with an emphasis on important clinical roles and provide insights into better integration of computational methods for clinical use.

**Abstract:**

Since the rise of next-generation sequencing technologies, the catalogue of mutations in cancer has been continuously expanding. To address the complexity of the cancer-genomic landscape and extract meaningful insights, numerous computational approaches have been developed over the last two decades. In this review, we survey the current leading computational methods to derive intricate mutational patterns in the context of clinical relevance. We begin with mutation signatures, explaining first how mutation signatures were developed and then examining the utility of studies using mutation signatures to correlate environmental effects on the cancer genome. Next, we examine current clinical research that employs mutation signatures and discuss the potential use cases and challenges of mutation signatures in clinical decision-making. We then examine computational studies developing tools to investigate complex patterns of mutations beyond the context of mutational signatures. We survey methods to identify cancer-driver genes, from single-driver studies to pathway and network analyses. In addition, we review methods inferring complex combinations of mutations for clinical tasks and using mutations integrated with multi-omics data to better predict cancer phenotypes. We examine the use of these tools for either discovery or prediction, including prediction of tumor origin, treatment outcomes, prognosis, and cancer typing. We further discuss the main limitations preventing widespread clinical integration of computational tools for the diagnosis and treatment of cancer. We end by proposing solutions to address these challenges using recent advances in machine learning.

## 1. Introduction

Cancer has historically been studied using genetic techniques, with the goal to identify gene-driver mutations that confer selective advantage and drive cells into a cancerous state. Driver mutations are distinguished from passenger mutations, which accumulate in the genome due to the changes undergone in the cancer cell as it becomes cancerous [1,2]. These approaches have led to several landmark discoveries and treatment successes, in particular, targeted therapies (Box 1) [3]. A prominent example is BRCA1/2 mutations in breast and ovarian cancers [4,5], which allowed for revolutionary treatment success for patients harboring the mutations by exploiting synthetic lethality (Box 1) through PARP inhibitors [6]. Identification of genes in the MAPK pathway, including BRAF and KRAS, has allowed for potent anticancer treatments in melanoma [7,8] and non-small-cell lung cancers [9,10]. IDH1 and IDH2 genes are inhibited in the treatment of AML [11] and gliomas [12], and ALK genes are targeted in lung cancers [13,14,15]. Furthermore, drugs targeting HER2 are a major treatment strategy for HER2-positive breast cancers [16,17,18,19,20,21]. However, most cancers are not driven purely by single-gene mutations; different genes or combinations of genes may confer a similar cancer phenotype. An understanding of how changes in multiple mutations or in the entire genome affect different cancers, and unraveling the biological sources of cancer mutations, has been a burgeoning field over the last decade [1,22].

Box 1 **Definition of select terms.**

Targeted therapiesTherapies targeting a specific protein associated with a diseaseSynthetic lethalityA type of interaction wherein a single event is tolerable but co-occurrence of two or more events is lethal Driver mutationA mutation that provides a selective advantage to a cell and transforms a cell into a cancerous statePassenger mutationA mutation that is a result, but not a direct cause, of a cell becoming cancerousMutagenic processAnything that causes damage to DNA or induces mutations in DNA, such as UV light, radiation, or alkylating agentsNon-negative matrix factorization (in progress)Unsupervised mathematical method wherein a single large nonnegative matrix is decomposed into two or more smaller matrices COSMICCatalogue of Somatic Mutations in Cancer: https://cancer.sanger.ac.uk/cosmic, accessed on 12 March 2023Signature 1Mutation signature associated with ageSignature 2Mutation signature associated with the mutagenic effects of APOBEC activitySignature 4Mutation signature associated with tobacco smokeSignature 7Mutation signature associated with UV exposureSignature 10Mutation signature associated with POLE proofreading errorsSignature 16Mutation signature associated with alcohol consumptionSignature 18Mutation signature associated with the mutagenic effects of the MUTYH geneDDRDNA damage repair, a network of processes that repairs damaged DNAMMRMismatch repair, a DDR pathway involved in detecting and repairing DNA mismatches BERBase-excision repair, a pathway that repairs typically small-scale mutations by first removing only the base and leaving an abasic site, which is later removed and replaced with other nucleotidesNERNucleotide-excision repair, a pathway that repairs mutations by entirely removing mutated sections of DNAHRHomologous recombination, a pathway repairing double-strand DNA damage that uses another strand of DNA as a template for repairNHEJNon-homologous end joining, a pathway repairing double-strand DNA damage that involves attaching two strands of broken DNA together. Logistic regressionA regression model for supervised classification LASSO logistic regressionA regression model that uses L1 regularizationRandom rorestAn ensemble machine-learning model that combines decision trees produced by baggingICIImmune-checkpoint inhibitors, a class of cancer drugs that suppresses pro-tumor immune-system regulatory effectsSupervised learningMachine-learning strategies wherein the classes of outcomes are knownUnsupervised learningMachine-learning strategies wherein the task of the model is to cluster the data into previously unidentified classes or discover the underlying classesNeural networkA machine-learning model that connects the input data to a desired output classification, where nodes connected by edges apply non-linear transformations to the data passed through the networkDeep learningMachine-learning models that are composed of multiple layers of neural networks stacked over one another (giving rise to the term “deep”) OverfittingFitting a particular data point too well and therefore failing to predict on other dataUnderfittingNot fitting the data well enough and inferring simplified decision rules that may not be optimized for any datasetGraph convolutional networks Neural-network architectures that represent graph data for learning tasks

## 2. Mutation-Signatures Background

**Figure 1:**
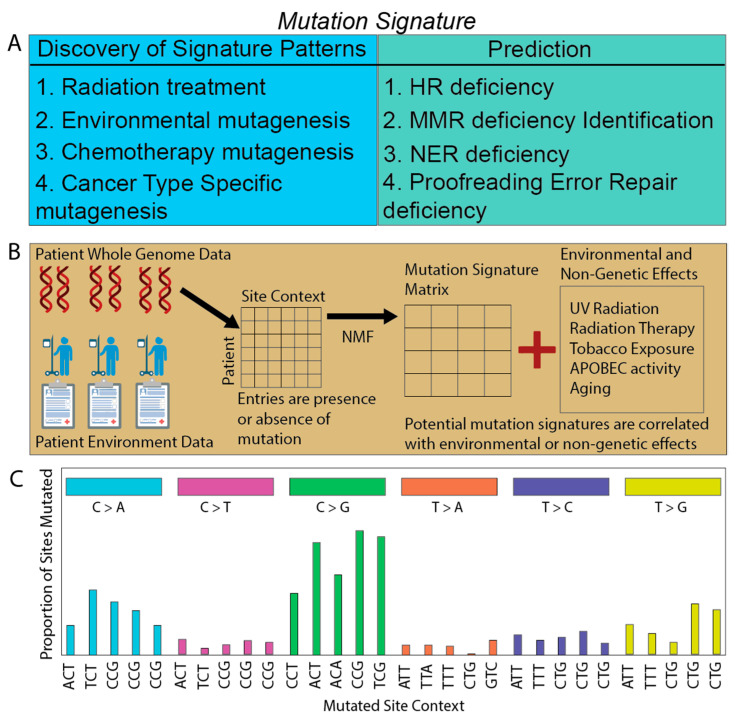
Mutation-signatures overview. (**A**) Mutation signatures have been used for discovery of genomic patterns reflecting the effects stressors have on the cancer genome and for phenotype prediction. (**B**) Simplified illustration of the construction of mutation signatures. Whole-genome-sequencing (WGS) data are collected and combined into a matrix. The matrix is decomposed using non-negative matrix factorization (NMF) or a similar method, and the resulting mutation-signature matrix is then correlated with environmental, patient-specific, or cancer-specific effects. (**C**) Simplified example of a potential mutation signature. The x-axis is site-specific nucleotide contexts. The colored boxes indicate groupings of the same nucleotide transition. The y-axis is the proportion of those context-specific sites that are mutated according to the specified transition. Only 30 of the 96 total potential sites are shown here for clarity.

One of the earliest and most important computational tools to uncover patterns of mutations arising through different mutagenic processes (Box 1) is mutation signatures, which triggered this revolution in understanding the holistic cancer genome (Figure 1). Mutation signatures were first developed by extracting patterns of nucleotide transitions within the mutations in whole-genome-sequencing data in a small cohort of breast cancers in 2012 [23]. In 2013, this principle was confirmed and expanded upon into a much larger dataset across different cancers [24]. These mutation signatures were a paradigm shift in understanding changes in the human genome in the context of cancer, as they allow patterns of historical mutations to be associated across the entire genome with biological, environmental, cancer-specific, and even cancer-treatment-specific effects (Figure 1A).

### 2.1. Deriving Signatures of Mutations

Mutation signatures mathematically model certain types of mutations that cluster together based on co-occurrence in tumors [24,25,26] (Figure 1B). The original types of mutation considered were based on nucleotide triplets [24]. Mutations were classified according to the transition from one base pair to another as defined from the pyrimidine of the Watson Crick base pair (6 potential transitions total, corresponding to C > A, C > G, C > T, T > A, T > C, T > G), as well as the nucleotide context of the surrounding two base pairs, yielding 96 total mutation types [24,26,27] (Figure 1C). The repertoire of mutation types considered has been subsequently expanded, including indels and double mutations, increasing the complexity and potential ability of the signatures to capture biological complexity across the genome [25,28].

Computationally, the first mutation-signature methods relied on the mathematical principle of non-negative matrix factorization (NMF) (Box 1), where a single large non-negative matrix is decomposed into two or more smaller matrices [29,30]. Multiplying these smaller matrices together should approximate the original input matrix. One of the decomposed matrices is the signature matrix representing the mutation signatures, which are, in a separate step, associated with outside environmental, cancer, or biological causes (see [26,27] or the supplemental information of [25] for a comprehensive mathematical explanation of mutation-signature generation, and in the first two, a comparison between different derivation methods). Subsequent checks are used to determine the optimal number of signature matrices. These include biological checks by investigating whether the cluster of mutations makes sense in the context of potential biological drivers and algorithmic checks, such as k-means clustering [24,25,26,27] (Figure 1B).

Many developments and refinements of the methods to generate mutation signatures have been suggested. Several rely on variations in NMF [24,25,31,32], but others use different methods to generate these signatures, resulting in potentially different signatures [26,27]. These methods include the NMF-based Sigprofiler [25], which is a newer version from the original mutation-signature paper, updated with more data [24], as well as the NMF-based MutSpec [31] and MutSignatures [32]. Additional methods are Bayesian NMF methods such as BayesNMF [33,34] and signeR [35]; probabilistic modeling, such as pmsignature [36] and EMu [37]; PCA-based methods, such as SomaticSignatures [38] and Helmsman [39]; and basic machine learning methods, such as deconstructSigs [40]. A recent comparison that evaluated the strengths and limitations of different methods for real and simulated data indicated that probabilistic models may perform better based on simulated data [27]. Others have developed methods to assess the reproducibility of the decomposition method itself [41], but comprehensive benchmarking is still needed. These signatures can be found in the Catalogue of Somatic Mutations in Cancer (COSMIC) (Box 1) [25], and other tools have been developed to allow for data analysis of mutation signatures [42,43].

### 2.2. Associating Mutation Signatures with Carcinogenic Processes

Once derived, the mutation signatures are then associated with potential biological, environmental, or cancer-related phenomena, and mutations that occur in these signatures may be extracted to investigate potential clinical relevance (Figure 1B). The landmark study by Alexandrov et al. (2013) established canonical mutation signatures that were used in numerous studies across the field and have been continuously expanded on by multiple laboratories. In the original study, age was associated with mutation-signature 1, later discovered by additional data to be two similar signatures labeled signatures 1A and 1B 1 (Box 1) and correlated with a C > T transition [24]. Age was associated with these signatures because the rate of mutation did not change across different ages and was consistent across cancers, indicating a steady baseline rate of mutation [24,25,28]. Subsequent work expanded upon using mutation signatures to track rates of mutations, which found that several signatures had clock-like processes associated with the passage of time but potentially varied across different tissues [28]. Signature 2 (Box 1) was associated with a family of cytidine-deaminase enzyme (APOBEC) activity, using previous work as a guide for the expected activity of APOBEC proteins [24,25,44,45]. Further work, seeking to investigate how mutation processes act in real time on live cells, confirmed signature 2 as being associated with APOBEC activity, and also found that APOBEC activity was sporadic, a finding that may have clinical opportunities and challenges when targeting mutagenic processes for treatment [46,47,48]. Another study investigating the cause of esophageal squamous-cell carcinoma found signatures associated with APOBEC activity, indicating activation of APOBEC was a driver in the formation of this cancer [49]. Individual genes may also be associated with certain mutation signatures. For example, germline mutations in the base excision-repair gene MUTYH left distinct mutation signatures corresponding to COSMIC signature 18 (Box 1) in colorectal cancers and adrenocortical carcinomas [50]. Mutation signatures have also been linked to known environmental carcinogens. Signature 4 (Box 1) mutations, which primarily involve C > A transitions on the transcribed strand, have been observed in lung, head and neck, and liver cancers and are associated with tobacco-smoke mutagens [24,25]. Studies confirming this association provided further evidence of smoking driving cancer by inducing genome-wide mutagenesis [51]. Another environmental association was found in the C > T transitions of signature 7 (Box 1), which was highly prevalent in melanoma, and indicated association with UV exposure [24,25]. Further incorporating indel mutations, multiple mutation signatures have been linked to diverse mutagenesis processes. These include substitution and indel-mutation signatures that correlated with mismatch repair and microsatellite instability in a subset of cancers [25,52]. Ionizing-radiation-mutation signatures, corresponding with single-nucleotide variations and indels, were identified in new cancer events of patients treated with radiation therapy [53]. Ionizing radiation can also interact with germline mutations to induce distinct mutation signatures, as demonstrated in TP53-deficient mice that were exposed to ionizing radiation [54]. Other environmental effects associated with mutation signatures include exposure to carcinogenic chemicals, including cobalt, vinylidene, and 1,2,3-trichloropropane. These associated effects were confirmed in both experimental mouse tumors and, in the case of 1,2,3-trichlorpropane, human tumors caused by contaminated drinking water [55]. Therefore, the analysis of thousands of cancer genomes allowed the delineation of various mutational signatures and some of these signatures to be linked to endogenous and exogenous mutagenic processes. Yet, the etiology of some of these signatures remains to be discovered.

## 3. Clinical Applications of Mutation Signatures: Promises and Challenges

**Figure 2:**
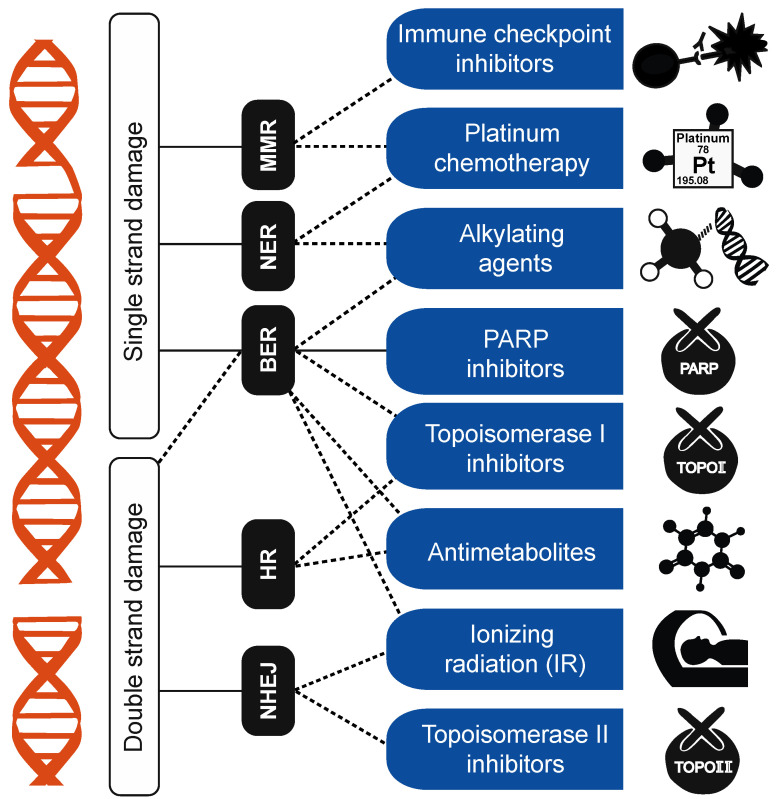
Potential clinical utility of genome-wide DNA-damage signatures: approved cancer drugs that induce DNA damage and associations with specific damage-repair pathways. DNA-damage-inducing drugs (lefthand blue boxes) activate DDR pathways (middle black boxes), directly or indirectly (solid and dashed lines, respectively). DDR pathways repair single- or double-strand damage, and impairment in those pathways leads to whole-genome signatures with potential clinical utility for DNA-damage-inducing drugs.

Concurrent with the development of mutation signatures was the recognition that these signatures may potentially be used in a clinical context for prognoses and treatment outcomes [23,24]. With their inherent ability to summarize genome-wide mutation patterns, mutation signatures are particularly useful when genome-wide mutagenesis is clinically relevant, or when genomic mechanisms modulating treatment outcomes are unknown (Figure 2).

### 3.1. DNA-Damage-Repair Footprints and Clinical Applications of Mutation Signatures

DNA damage repair (DDR) is a complex network comprising multiple DNA-repair pathways, damage-tolerance processes, and cell-cycle checkpoints, with multiple interacting components assessing and maintaining genomic integrity [22,56,57]. Impairment of DDR components leads to genomic instability, a central characteristic of almost all human cancers [58,59]. Several forms of genomic instability have been found in tumors and associated with different DDR pathways [59]. Single-strand DDR pathways include mismatch repair (MMR) (Box 1), base-excision repair (BER) (Box 1), and nucleotide-excision repair (NER) (Box 1). Impairments of these mechanisms lead to genome-wide accumulation of base-pair mutations, involving base substitutions, deletions, or insertions of a few nucleotides, as well as local copy-number amplifications and deletions [56]. Homologous recombination (HR) (Box 1) and non-homologous end joining (NHEJ) are double-strand DDR pathways correcting DNA double-strand breaks (DSBs), which can lead to genomic imbalances and translocations [57,60,61].

Disruption in DDR pathways induces genome-wide mutagenesis, and some DDR pathways are linked to responses to specific treatments, including chemoradiation and targeted therapies. Mutation signatures become useful in such cases, as they can examine patterns of DDR deficiencies throughout the genome.

This concept has been most clearly shown in applications to HR-deficient cancers. Loss of HR results in increased sensitivity to inhibition of the BER gene PARP1. The absence of PARP allows for unrepaired single-strand breaks to accumulate, and these breaks collide with replication forks and induce cytotoxic double-strand breaks. When HR deficient, cells are unable to repair those breaks, leading to genomic instability and cell death [62,63]. Therefore, strategies to infer HR deficiency in tumors are particularly useful for treatments targeting HR-deficient cells. One important tool developed to identify HR deficiencies in breast cancer is HRDetect, which is based on a LASSO logistic-regression model (Box 1) that uses mutation signatures associated with substitutions, indels, and rearrangements as feature inputs to the model [64]. Subsequent analysis showed that this tool was able to identify HR repair-deficient patients (HRD) irrespective of their HRD germline, genetic, or epigenetic status [65,66]. HRDetect was also shown to potentially be able to identify patients that would respond to platinum treatments [67]. The benefit of HRDetect and similar tools is the identification of patients that are sensitive to PARP inhibitors or platinum treatment but that could be missed in the traditional HR-deficiency screen [33,64,67,68,69]. HRDetect was used in a secondary endpoint of a phase II clinical trial examining PARP inhibitors for triple-negative breast-cancer patients, with success in identifying HR-deficient tumors that could be missed using current clinical practice [69]. Recently, other tools have also been developed to detect HR deficiencies using mutation signatures, including CHORD and SigMA, which use a random-forest (Box 1) and likelihood-based approach (Box 1) to classification, respectively [68,70].

Other treatments targeting HRD cancers are currently in clinical trials, where mutation signatures may become useful. These treatments target different proteins involved in the HR pathway, for example, ATR inhibitors [71]. ATR inhibitors (ATRi) may selectively kill HRD cells [72]. ATR-induced cell death has also been shown in PARP-resistant cancers, indicating the complementarity of this approach with PARP [73,74]. ATRi for treatment of HRD cancers is currently in clinical trials [75]. Therefore, models using mutation signatures could also provide a way to identify patients that would benefit from ATRi therapy.

Mutation signatures can also infer MMR deficiencies (MMRd). Importantly, MMRd is an approved biomarker for immune-checkpoint inhibitors (ICI) (Box 1) [76], and similar to HR deficiencies, MMRd leaves distinct mutational footprints on the genome. MMRDetect is a tool developed to infer mutation signatures descriptive of MMRd using a logistic-regression model (Box 1) incorporating mutation signatures associated with MMRd (Table 1) [77]. Although direct sequencing of potential causal genes (such as *MSH2*, *MSH6*, *PMS2*, and *MLH1*) are clinically available for MMR [78,79], research has shown that these genes may potentially be epigenetically regulated rather than genetically mutated [80,81], posing a challenge for MMRd detection through genomic screening. Analyzing the effects of MMR across the genome using mutation signatures could complement identification of cancers deficient in MMR that may be susceptible to certain treatments. These treatments primarily involve immune-checkpoint-inhibitor therapy, but recent work demonstrated that inhibiting Werner helicases in MMRd tumors may induce synthetic lethality and potentially allow for additional treatment options [78,82,83] Further supporting this notion, studies carried out in pancreatic cancer found associations between MMR signatures and antitumor immune activation, even when canonical HR or MMR genes were not germline mutated in the tumors (Table 1) [84].

Other associations between cancer treatments and distinct DDR pathways include ERCC2 helicase in the NER pathway (Table 1). Mutated ERCC2 produces a distinct mutational signature that serves as a marker for disruption in the NER pathway [34]. Mutation signatures corresponding to NER patterns similar to ERCC2 disruption could provide a biomarker for cisplatin or similar platinum treatment [34,85].

Other than canonical DDR pathways, proofreading errors also induce distinct mutation signatures, potentially allowing for the development of similar methods to MMR and HR mutation-signature tools. For example, POLE proofreading errors are associated with Signature 10 (Box 1), which could be associated with immune-checkpoint-inhibitor therapy sensitivity (Table 1) [86,87]. Overall, the link between specific DDR pathways and mechanisms or sensitivity of distinct cancer treatments warrants more work exploring this association through mutation signatures.

### 3.2. Mutation Signatures as Clinical-Discovery Tools

Due to their ability to elucidate associations between exogenous or endogenous mutagenesis and cancer, mutation signatures are useful for studying clinical phenomena when the underlying mechanisms and genetic markers are unknown. Therefore, these signatures may be useful for clinical development and discovery (Figure 2).

Radiation therapy has long been recognized as a potential driver of new cancers [99,100], but markers distinguishing radiation-induced tumors are unknown. Mutation signatures have been used to differentiate cancers driven by radiation therapy as opposed to cancer relapse or recurrence (Table 1) [53]. Another study applied mutation signatures to identify an association between TP53 deficiency and radiation-induced secondary cancers in mice (Table 1) [54]. Similarly, a potential association with radiation and mutation signatures was found in mutation-signature ID12, with higher mutation-signature activity in HRD tumors compared to non-HRD tumors (Table 1) [88]. Therefore, mutation signatures have been useful for identifying patterns linked with a distinct mutation that in turn may be used as a marker for patients that should not be treated with radiation therapy.

Mutation signatures are being used to investigate the effects of other cancer treatments on the genome, allowing both a better understanding of the mechanism of the treatments and potential indications or contra-indications of the treatment. For example, using mutation signatures, 5-FU was found to induce numerous T > G substitutions throughout the genome, indicating a potential tumorigenic effect of this chemotherapy drug (Table 1) [89]. Further work has also shown mutation-signature associations with platinum therapies and capecitabine and confirmed 5-FU associations, with increasing time and doses of drugs producing higher mutation-signature signal (Table 1) [88].

Mutation signatures have also driven discovery of clinically relevant environmental carcinogens through patterns of mutations in the genome. Aristolochic acid (AA) is a chemical found in plants used in herbal remedies. In different cancers, and in bladder cancers in particular, the presence of AA-associated signatures provided evidence that AA has a mutagenic effect on the genome, demonstrating the potential of mutation signatures as a screening tool (Table 1) [90,91,92]. Evidence from several studies on esophageal squamous-cell carcinoma also found associations between alcohol consumption and several mutation signatures [93,94]. Specifically, mutation signature 16 (Box 1), associated with alcohol consumption, was also present in liver cancers [95]. Similarly, a study across many different cancers found a distinct mutation signature associated with alcohol consumption in HNSC, ESCA, and LIHC and proposed a mechanism of mutation involving acetaldehyde (Table 1) [96]. These and similar signatures summarizing cancer-risk factors may inform patients and possibly be developed into screening practices.

Another promising use of mutation signatures is as a biomarker for different cancer types or cell types. Mutation signatures were used to distinguish different cell types within esophageal adenocarcinoma, with the potential to directly target these different subtypes for different therapy treatments (Table 1) [97]. Recent work has also shown that distinct patterns of mutation signatures combined with additional tumor information can be used with machine learning to identify secondary tumors of unknown primary, which can greatly facilitate targeted treatment of the cancer (Table 1) [98].

The clinical potential of mutation signatures in other contexts has been mentioned in multiple studies, for example, for predicting immunotherapy response [1,86,87]. In practice, however, mutation signatures have so far demonstrated clinical utility as a biomarker only when whole-genome changes reflect the outcome of interest or as a tool for clinical discovery when underlying mutagenic processes are unknown. In clinical practice, summarizing a mutagenic process to a defined set of genes or markers is both more interpretable to clinicians and requires sequencing fewer genomic regions. Therefore, mutation signatures are useful in the path to defining mutagenic processes and finding associated markers to be used in the clinic.

## 4. Beyond Mutation Signatures: Computational Approaches to Infer Clinically Relevant Patterns of Mutations

**Figure 3:**
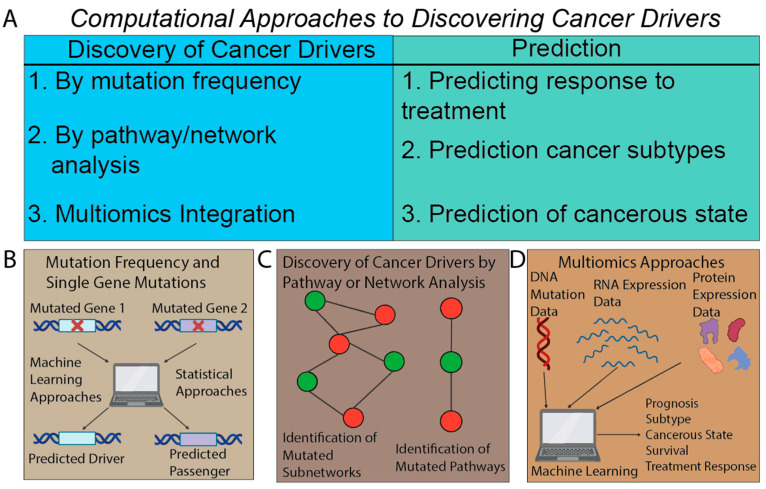
Overview of computational approaches for using mutational patterns beyond mutation signatures. (**A**) Computational approaches are used for discovery of cancer-driver mutations and for the prediction of cancer phenotypes using mutational patterns. (**B**) Single gene for distinction of cancer-driver mutations. (**C**) Network- and pathway-based methods to predict driver mutations and use mutational data for cancer-phenotype prediction. (**D**) Multi-omics approaches integrate mutations with different data types to improve discovery of cancer drivers and prediction of cancer phenotypes.

In addition to mutation signatures, other methods have been developed to discover patterns of cancer mutations that drive cancer development and underlie clinical outcomes (Figure 3). The majority of these methods derive patterns of mutations using supervised- or unsupervised-learning strategies (Box 1), which can then be directly correlated with a clinical outcome of interest (Figure 3A). A fundamental goal of these emerging techniques is the identification of cancer drivers. Discovering mutated genes that are drivers of tumorigenesis and distinct from genes that are merely passengers is essential to understanding cancer development and finding the causal players that may be clinically targeted [101]. Therefore, a comprehensive catalogue of driver mutations can improve diagnosis and prognosis and provide for new drug targets [102,103]. In recent years, as sequencing data has become increasingly available, several methods have been developed that use machine-learning techniques to distinguish potential driver mutations from passenger mutations (Figure 3B). These methods have steadily advanced to incorporate different aspects of the genome. Early work in this field involved developing methods analyzing the frequency of mutations in genes within cancers to separate out potential driver genes from passengers, such as MutSigCV [104], inVex [105], and MuSiC [106]. Later approaches incorporated functional impact by predicting the changes to the amino acids linked to a mutation and predicting the impact of a mutation to the function of a gene. Such tools include the random-forest-based CHASM [107,108,109], polyphen2 [110], e-Driver [111], and SIFT [112,113], which were adapted to cancer mutations. Taking this functional concept further, other algorithms use the structure of the protein itself to predict relevance to cancer. These include MSEA [114], which combines mutation frequency and protein-domain structure to predict driver genes, and iPAC [115] or GraphPAC [116], which use tertiary structure to predict driver mutations. More specialized methods such as ActiveDriver [117] have focused on mutations in phosphorylation or similar post-translational regulation sites (Table 2).

Methods have also shifted from focusing on features of single genes to accounting for more complex patterns, such as gene networks and pathways (Box 1) (Figure 3C). These approaches seek to leverage the knowledge that genes do not operate in isolation but act as part of a larger whole, where mutations in similar pathways or network locations may produce similar effects. For example, HotNet2 uses a heat-diffusion model to identify mutated subnetworks, providing more information about the mutational landscape than mutation data alone [118]. This work allowed for the identification of rare driver mutations in the TCGA compared to previous studies focusing on purely mutation-based analysis. Other network approaches include MUFFIN, which used the mutation data in network neighbors to discover cancer drivers, even with a subset of the data [119], and Paradigm, which used curated pathways with a gene-factor graph-modeling approach to discover cancer drivers (Table 2) [120]. Newer methods have expanded on this network-based analysis to discover modules of tumor–gene interactions with potential diagnostic and therapeutic significance [121] and have also incorporated non-coding mutations, pathways, and network analysis [122]. Beyond network or pathway analysis, a recent study developed a deep-learning model (Box 1) for the background mutation rates to identify patterns of positive selection and find driver mutations in coding and non-coding regions [123]. Another method, boostDM, combined mutational data across cancers with gradient-boosting tree algorithms (Box 1) to produce a series of interpretable models for the identification of cancer drivers, and it has even been reported that this method outperforms experimental large-scale saturation-mutagenesis experiments (Table 2) [124]. A recent benchmarking and comparison of these methods found that four methods were most effective at predicting drivers [125], namely, the random-forest-based CHASM [107,108,109] and DEOGEN2 [126], the PCA-based CTAT-cancer [127], and the deep residual neural-network-based PrimateAI [128] (Table 3).

Other than discovery of driver mutations, methods have used pathway and network information to identify patterns of mutations to predict treatment outcomes, allowing for more biologically interpretable models (Table 2) [129,130]. An early representative study used network-based stratification to combine mutation data and gene networks to predict patient responses, tumor types, and histology [131]. A method to de-novo identify significantly mutated subnetworks has revealed known and new mutated pathways in cancer. Mutation data aggregated into biological processes were used as input to different machine-learning classifiers to predict immunotherapy response in melanoma and to understand biologically what occurs in immunotherapy response and resistance [132]. Pathway-based methods have also been developed for scoring responses to different cancer treatments, showing applications in both drug discovery and clinical selection of drugs [133]. Pathways and mutation data were also used to identify cancer subtypes and prognostic indications of several of those subtypes [134]. In another study, mutated pathways were correlated with different DNA-damage-response mechanisms to detect tumors mainly associated with aneuploidy and those with defective DNA repair or microsatellite instability, thus identifying groups of mutated genes that predict patients’ outcomes [135]. Recent work using deep learning has used pathway information, mutations, and copy-number variation to predict patient response to immunotherapy in melanoma [136]. An important benefit of these pathway-based approaches is an emphasis on biological interpretation of predictions, which are often considered more important than model performance (Table 2) [137].

Mutations in a single gene or within a specific pathway may not be sufficient for characterizing cancer development or clinical outcomes. More complex patterns and interactions between mutations confer more information for clinical-prediction tasks. Methods to identify combinations of mutations were used to distinguish tumors from healthy tissues [138], to find patterns of mutually exclusive mutations [139,140] and epistasis [140,141], and to predict patient survival and immunotherapy benefit (Table 2) [142]. Somatic mutations were analyzed by unsupervised NMF and supervised machine-learning methods to predict breast-cancer subtypes, with potential therapeutic significance [143]. Combinations of passenger mutations were recently used in a deep-learning neural network to classify metastatic tumors of unknown origin [144], and found that passengers conferred more information for predicting the tissue of origin. Some computational methods identified mutation patterns to infer the order of mutations in tumor evolution [145,146,147,148,149] or used timing of mutations [150], clonality [151,152], and machine-learning models [153] to predict clinical outcomes (Table 2, Table 3).

Some methods have incorporated tumor mutations with other types of data to predict response to cancer therapies (Table 2, Table 3, Figure 3D) [154]. For instance, in breast cancer, patient response or resistance to paclitaxel or gemcitabine was predicted using SVM models applied to gene mutations, copy number, and expression [155]. This study found that the mutation data alone were not sufficiently informative, likely due to sparsity. Studies have also incorporated genomic and transcriptomic information to predict ICI response and extract clinically relevant targets using a logistic-regression model [156]. Mutation data were incorporated with gene-expression-based diagnostic models to correlate clinically relevant mutations with gene-expression patterns in HCC, allowing for the identification of HCC cells compared to normal liver cells [157]. Other work has used multiomics integration of mutations and other data types with interaction and pathway information to predict ovarian-cancer outcomes [158]. A multiomics approach incorporated mutations, transcription information, epigenetics, and drug targets in a deep-learning framework to predict drug repurposing for cancer treatment [159]. Mutations in specific driver genes were also included in a multiomics integration through deep learning to predict survival in liver-cancer cases [160]. Multiomics integration has also been used to predict TMB in lung-cancer patients, which may potentially be clinically relevant for predicting response to immunotherapy in many cancers (Table 2) [161]. However, the clinical utility of multiomics integration has not been fully demonstrated, where limited amount of complex data is a serious bottleneck for development of computational methods to infer clinically relevant multiomics patterns [162].

**Table 2:** Methods inferring clinically relevant mutation patterns beyond mutation signatures.

Task Category	Sub-Category	Clinical Relevance	Example Methods
Identifying cancer drivers	Cancer drivers by mutation frequency	Cancer-driver discovery; Obtaining cancer drivers for prognosis, cancer identification, and treatment	Methods based on mutation frequencies: MutSigCV [104], Invex [105], Music [106]
			Amino-acid and functional-impact changes: Chasm [107,108,109], polyphen2 [110], SIFT [112,113]
			Protein structure: MSEA [114], iPACT [115], GraphPac [116]
			Phosphorylation-site mutation: ActiveDriver [117]
	Cancer drivers by pathway		Heat diffusion: HotNet2 [118]
			Mutated neighbors: MUFFIN [119]
			Curated pathways and gene-factor modeling: Paradigm [120]
			Network-based modules [121]
			Network-based coding and non-coding modules [122]
			Deep-learning cancer-driver analysis [123]
			Computational-saturation mutagenesis [124]
Exploring mutated pathways	Predicting outcomes using pathways	Patient-prognosis prediction	Identification of genes associated with DNA-damage response and clinical outcomes [135]
		Patient response to immunotherapy	Machine learning on clinical mutation data to predict patient response to ICI in melanoma and other cancers [132]
			Deep learning on pathway information, mutations, and copy-number variation to predict melanoma outcomes [136]
		Detecting drug targets through pathways	Drug discovery and tailored treatments [133]
	Pathways of cancer subtypes	Cancer-subtype identification	Cancer-subtype identification and prognosis [134]
		Prediction of patient response, tumor type, and histology	Gene-network-based stratification using mutation data for prediction [131]
Identifying complex patterns of multiple mutations	Inferring interactions between mutations	Interactions conferring sensitivity	Mutual-exclusivity analysis of genes [139,140]
			Epistatic effects of genes [140,141]
	Clustering samples	Cancer-type identification	Unsupervised NMF and supervised ML to identify cancer subtypes [143]
			Applying deep-learning neural network to passenger mutations to classify metastatic cancers of unknown origin [144]
		Identification of tumors vs. healthy tissues	Gene-combination analysis [138]
	Inferring order of mutations	Inferring timing of mutations	Mutation patterns to infer order of mutation events [145,146,147,148,149]
		Determining timing for predicting clinical outcomes	Mutation timing to predict clinical outcome [150]
			Clonality analysis for outcome prediction [151,152]
			Machine learning to predict outcome through mutational time series [153]
Multiomics approach: integrating mutations with other data types	Multiomics outcome prediction	Chemotherapy response or resistance	Using SVM on mutations, copy number, and expression for chemotherapy prediction [155]
		ICI response or resistance	Genomic and transcriptomic information for response or resistance to ICI [156]
		Prediction of patient outcomes	Mutation, interaction, and pathway information to identify ovarian-cancer outcomes [158]
		Mutation-burden prediction for ICI therapy	Lung-cancer mutation-burden prediction using a multiomics approach [160]
	Cancer classification	Identification of cancerous vs. non-cancerous cells	Identification of HCC cells from normal cells through mutation and expression information [157]
	Identification of drug targets	Drug repositioning	Mutations, expression, epigenetics, drug targets, and deep learning for drug repositioning [159]

## 5. Major Challenges for Clinical Utility of Complex and Data-Driven Mutational Patterns

**Figure 4:**
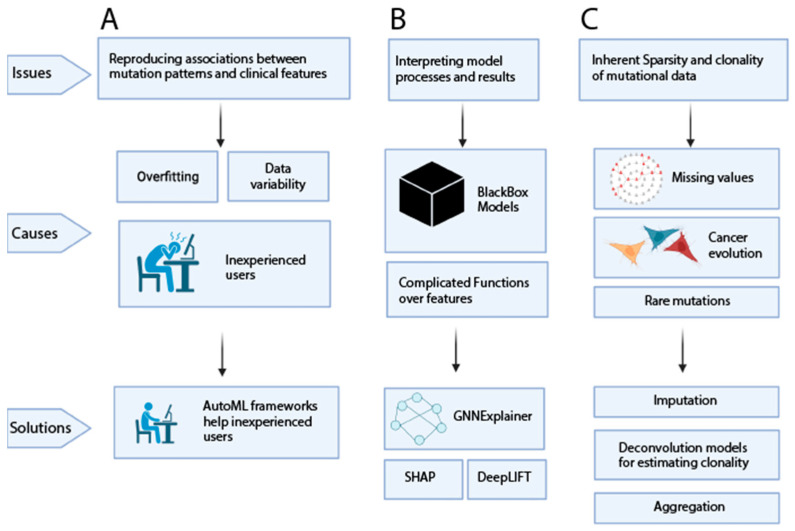
Key challenges for clinical integration of computationally derived mutational patterns and machine-learning methods that address these issues, including (**A**) reproducibility, (**B**) interpretability, and (**C**) inherent sparsity and clonality of the mutational data.

Despite substantial efforts to identify clinically relevant cancer mutations and patterns, complex patterns beyond single-gene mutations have not been integrated into the clinic. There are several challenges for computational approaches that have prohibited the clinical success of data-driven mutational patterns, which are outlined below, along with potential ways forward to overcome these challenges (Figure 4).

A major and fundamental challenge to overcome is the difficulty of recapitulating associations between mutational patterns and clinical features across multiple studies (Figure 4A) [163,164]. This issue of reproducibility is especially pertinent in the context of clinical significance [165]. Reproducibility issues can result from model underfitting or overfitting (Box 1) due to biological or clinical confounders, small sample settings, data sparsity, or noisy and variable data [166,167]. Both under and overfitting result in failure to generalize findings to other studies, and failure to establish clinically useful biomarkers. Other factors that can lead to unreproducible results are errors and poor documentation of code and data processing [168] and lack of availability of the software and methods used [169]. With multiple parameters and intricate biological datasets, even in well-documented studies, it can be very difficult to fully reproduce results [164]. More complex model and mutation patterns may improve the performance but also risk overfitting. It is therefore important to follow guidelines and tools for reproducible computational work [170,171]. To ensure reproducibility with an eye to clinical integration, correct training, validation, and testing practices in machine learning should be followed, along with standardized methods, automation, transparency, and good coding practices [172,173,174]. Studies should also ensure generalization across different, biologically independent datasets [175,176,177].

Tools are also being developed to assist non-specialists with ML applications (Table 3). One example for such tools are automated machine-learning (AutoML) pipelines, which handle the required tasks of applying machine learning to user-provided datasets. In recent years, several frameworks that handle hyperparameter optimization and model selection have become available [178,179,180,181,182,183,184,185]. Such frameworks can also be adapted by non-expert machine-learning users in biomedicine, which can help support reproducibility for machine-learning applications. Beyond the model itself, failure to reproduce results can also be caused by poor laboratory or data-handling practices, human error such as mislabeling, or contaminators, among other sources of variability [186,187].

Another important challenge to overcome in the path to clinical integration is the issue of biologically interpretable results (Figure 4B) [113,137,188]. An interpretable model allows for an understanding of the data that go into the model, the processes applied by the model, and of how the model arrives at the results [189,190,191]. This is important because clinicians and biologists typically favor biological interpretability over black-box models [192,193], even at the expense of the predictive capability of the model. An interpretable model can also provide for follow-up biological discoveries and a better understanding of unexpected results [194]. More complex models or patterns that may demonstrate better performance are likely to be less interpretable. For example, cancer-driver identification is complex, and increasingly more sophisticated models have been developed to address this complexity, but even more complex models have not necessarily expanded on the drivers being discovered.

To address this complexity, many interpretation approaches have been proposed to provide explanations for the trained models’ predictions and the features driving the model to make a specific prediction (Table 3). LIME is a popular interpretation tool that learns a new interpretable model that can better explain a less interpretable model. Numerous studies have successfully applied LIME to provide interpretation of complex models, including in biomedicine [195,196]. Another popular interpretation method is DeepLIFT [197], which calculates the contribution of neurons in a trained neural network by evaluating the difference in activation from a chosen representative reference. DeepLIFT has also been useful for interpreting model prediction in genomic datasets [198,199,200,201,202,203]. Another interpretive model is SHapley Additive exPlanations (SHAP) [204], which is based on the Shapley value from game theory. This method generates contribution values called SHAP values for each feature, which represents the differences between the actual prediction and the expected prediction of a trained model. SHAP values not only provide insight into how much each feature contributes to the prediction but also to the direction of the contribution, either towards the positive class or the negative class. Multiple biomedical studies have used SHAP to provide clear explanations of features driving predictions [124,205,206,207,208,209].

Another form of explanatory methods is through biological-network explanations (Table 3) [210,211]. Biological networks have been used to build network-based predictive models based on graph convolutional networks (GNN) (Box 1) [212,213,214]. An interpretability challenge for a GNN learning biological networks is understanding the network structure and how sub-networks contribute to the prediction. GNNExplainer [215] provides explanations of GNN-based prediction by identifying a dense sub-network structure along with a small subset of node features that play an important role in the GNN-based prediction. GNNExplainer can be used to understand the contributions of sub-networks’ nodes and their roles in determining predictions, allowing for biological interpretability. Interpretation models can help bridge the gap between model developers and clinicians, potentially allowing for clinical utility of more complex model-based mutational patterns [190,191,216,217,218].

Another challenge for uncovering complex patterns of mutations is linked to the sparse nature of mutation data themselves (Figure 4C). Mutations, even in cancer, are generally infrequent when the entire genome or exome is taken into consideration [219,220]. This sparsity extends to other sources of biological data [220,221,222,223,224,225]. Most machine-learning models have difficulty learning and picking up patterns for prediction based on sparse data, which can lead to overfitting [219,226,227,228,229,230]. This results in poor reproducibility [227,228,229,230]. Feeding into this issue is the fact that cancer is highly heterogeneous, and rare events do not preclude clinical relevance [231,232]. Including the methods discussed above, aggregation of mutations can potentially mitigate this sparsity. Although aggregation may reduce sparsity, care must be taken to ensure results are biologically interpretable [233,234]. 

Another factor that can lead to sparsity is missing data. Missing data can result from experimental design or different types of human errors [235,236]. In addition to sparseness, missing data can also lead to biased datasets and results [237]. Several techniques have been developed to handle missing data, such as imputing missed instances with estimated values [238,239,240]. Machine-learning methods can also be used to perform data imputation, such as regression- and ensemble-based models [241,242]. Furthermore, several methods have been developed recently to improve the quality of data imputation [243,244,245,246,247].

Another challenge is linked to how mutations are accumulated in cancer evolution. As cancer develops, mutations arise in certain cell lineages, and tumor mutations are therefore clonal and not homogenous [248,249,250]. Different cells within the same tumor have different clonal lineages and therefore different patterns of mutations [248,251]. Within the same patient, lineages can be very different. This complicates typical data analysis because the data being analyzed are subjected to specific clonal lineages where some mutations may be misrepresented. As a result, in bulk datasets the actual clinically relevant mutational players may be obscured [252] and the clonal composition of the tumor may change over time, especially in response to treatment. Several methods have been developed to address issues surrounding clonality [251,253,254,255,256,257], but more work is needed to address clonality in the context of computational tools and modeling.

## 6. Summary

With the introduction of next-generation sequencing, numerous causal and actionable mutations have been identified and used clinically as biomarkers or for new targeted therapies. Due to the increasing realization of the vast complexity underlying tumorigenesis, future clinical breakthroughs are likely to increasingly rely on computational methods to identify these clinically actionable patterns of mutations. Mutation signatures allow for exploration of intricate patterns of mutations in cancer, effectively identifying mutational patterns to describe DDR pathways and environmental effects. However, mutation signatures require extensive sequencing of cancer genomes, limiting clinical applications beyond these purposes. Other methods have been developed to uncover complex patterns of mutations for clinical use. These include methods that identify drivers of cancer, methods that predict clinical outcomes by integrating mutations with biological pathways, and methods incorporating other types of omics. However, such methods have yet to be integrated into the clinic. The major challenges for clinical integration of computationally driven mutational patterns are lack of reproducibility, the difficulty of interpreting complex models, and issues associated with intrinsic attributes of cancer-mutational data, such as sparsity and clonality. State-of-the-art computational and machine learning can be adjusted to address these issues, improving the interpretation of complex models and enhancing reproducibility. With the consistent accumulation of cancer-genomic datasets and the complexity of cancer genomes, many of the next great clinical breakthroughs in cancer research will rely on computational tools to fully understand the complicated patterns of mutations that characterize cancer.

**Table 3:** Summary of tools reviewed in this article, software resources, and mention of the review section where tools are referenced. All referenced tools and websites were accessed between 16 February 2023 and 19 February 2023.

Method Name	Method Description	Code/Tool	Reference	Review Section
IntOGen	A method to access the database of mutational-cancer drivers	https://www.intogen.org/search	[2]	1
SigProfiler	Framework for deciphering mutation signatures from mutational catalogues of cancer genomes	https://www.mathworks.com/matlabcentral/fileexchange/38724-sigprofiler	[24,25,26,27]	2.1
MutSpec	Somatic-mutation analysis in human and mouse	https://toolshed.g2.bx.psu.edu/	[31]	2.1
MutSignatures	Cancer-mutation-signatures analysis	https://github.com/dami82/mutSignatures	[32]	2.1
SigneR	Bayesian approach to discover mutation signatures	http://bioconductor.org/packages/release/bioc/html/signeR.html	[35]	2.1
pmsignature	Probabilistic model to infer and visualize cancer-mutation signatures	https://github.com/friend1ws/pmsignature https://friend1ws.shinyapps.io/pmsignature_shiny/	[36]	2.1
SomaticSignatures	Inferring characteristics of mutation signatures	https://www.bioconductor.org/packages/release/bioc/html/SomaticSignatures.html	[38]	2.1
Helmsman	Mutation-signature analysis	https://github.com/carjed/helmsman	[39]	2.1
deconstructSigs	Mutation signature by machine learning	https://github.com/raerose01/deconstructSigs	[40]	2.1
SignatureEstimation	Discovering the existence of mutation signatures in cancer	https://www.ncbi.nlm.nih.gov/CBBresearch/Przytycka/index.cgi#signatureestimation	[41]	2.1
Signal	Mutation-signature analysis	https://github.com/Nik-Zainal-Group/signature.tools.lib	[42]	2.1
MutationalPatterns	Comprehensive analysis of mutation processes across the genome	http://bioconductor.org/packages/release/bioc/html/MutationalPatterns.html	[43]	2.1
	Identification of mutation signatures	https://github.com/team113sanger/mouse-mutatation-signatures	[55]	2.2
CHORD	Classifier identifying homologous recombination deficiency across cancers	https://github.com/UMCUGenetics/CHORD	[68]	3.1
SigMA	Identification of mutation signatures	https://github.com/parklab/SigMA	[70]	3.1
mutfootprints	Identification of mutation footprint of and for cancer treatment	https://bitbucket.org/bbglab/mutfootprints/src/master/	[88]	3.2
	Identification of mutation signatures	https://github.com/UMCUGenetics/5FU	[89]	3.2
CUPLR	Classification of primary-tumor identity of metastatic tumors	https://github.com/UMCUGenetics/CUPLR	[98]	3.2
MutSigCV	Identification of mutated genes in cancer	https://software.broadinstitute.org/cancer/cga/mutsig	[104]	4
inVex	Identification of positive selection for non-silent mutations	https://software.broadinstitute.org/cancer/cga/invex	[105]	4
MuSiC	Identification of mutational relevance in cancer genome	http://gmt.genome.wustl.edu/	[106]	4
CHASM	Identification of important biological single-nucleotide mutations in cancer	http://wiki.chasmsoftware.org/index.php/Main_Page	[107,108,109]	4
PolyPhen-2	Classification of missense-mutation damaging effects on protein	http://genetics.bwh.harvard.edu/pph2/	[110]	4
e-Driver	Identification of protein functional regions driving cancer	https://github.com/eduardporta/e-Driver	[111]	
SIFT	Classification of amino-acid-substitution impact on proteins	https://sift.bii.a-star.edu.sg/	[112,113]	4
MSEA	Classification of cancer genes based on patterns of mutation hotspots	https://github.com/bsml320/MSEA	[114]	4
iPAC	Identification of non-random somatic mutations in proteins	http://www.bioconductor.org/packages/2.12/bioc/html/iPAC.html	[115]	4
GraphPAC	Identification of non-random somatic mutations in proteins	http://bioconductor.org/packages/release/bioc/html/GraphPAC.html	[116]	4
ActiveDriver	Effect of mutation on post-translational signaling	http://www.baderlab.org/Software/ActiveDriver	[117]	4
HotNet2	Identification of rare somatic-mutation combinations in pathways and protein complexes	http://compbio-research.cs.brown.edu/pancancer/hotnet2/#!/ http://compbio.cs.brown.edu/software/	[118]	4
MUFFINN	Cancer-gene detection through network analysis of somatic mutations	http://www.inetbio.org/muffinn/	[119]	4
boostDM	Identification of driver mutations in cancer genes from observed mutations in human tumors	https://zenodo.org/record/4813082#.Y9L38dLMKV4	[124]	4
DEOGEN2/MutaFrame	Classification of single-amino-acid variant loss in human proteins	http://babylone.3bio.ulb.ac.be/MutaFrame/	[126]	4
PrimateAI	Classification of clinical impact of human mutations	https://basespace.illumina.com/s/cPgCSmecvhb4	[128]	4
	Classification of immune-checkpoint-inhibitor therapy response	https://github.com/AuslanderLab/Mutated_pathway_ICI_prediction	[132]	4
	Identification of associations between driver mutations and chromosomal aberrations	https://github.com/noamaus/INTERPLAY-TUMOR-CODES	[135]	4
KP-NET	Classification of immunotherapy response	https://github.com/0219zhang/KP-NET	[136]	4
	Causal identifications of individual instances of cancer	https://bitbucket.org/sajal000/multihit-combinations/src/master/	[138]	4
CLICnet	Identification of somatic-mutation combinations that predict cancer survival	https://github.com/gussow/clicnet	[142]	4
	Classification of primary and metastatic tumors	https://github.com/ICGC-TCGA-PanCancer/TumorType-WGS	[144]	4
SMASH	Identification of somatic-mutation associations	https://github.com/Sun-lab/SMASH	[152]	4
	Learning evolution of a tumor through mutational time series	https://github.com/noamaus/LSTM-Mutational-series	[153]	4
	Classification outcomes of checkpoint inhibition by tumor and immune-signal combination	https://zenodo.org/record/5528497#.Y9Ps1dLMKV4	[156]	4
DeepDRK	Drug response prediction	https://github.com/wangyc82/DeepDRK	[159]	4
MetAML	Prediction of metagenomics-based tasks	https://github.com/segatalab/metaml	[176]	5
	Generalization in machine learning for dataset characteristics	https://github.com/pietrobarbiero/dataset-characteristics	[177]	5
Auptimizer	Hyperparameter optimization	https://github.com/LGE-ARC-AdvancedAI/auptimizer	[178]	5
TPOT	Automated ML–tree-based optimization pipeline	https://github.com/EpistasisLab/tpot	[181,182]	5
Hyperband	Hyperparameter optimization	https://github.com/automl/pylearningcurvepredictor	[183]	5
DanQ	Classification of the function of DNA de novo mutations from sequences	http://github.com/uci-cbcl/DanQ	[188]	5
	An explainable machine learning tool of severity-level predictions of COVID-19 patients	https://github.com/freddygabbay/covid19explainableML	[196]	5
DeepLIFT	An explainable machine-learning tool	https://github.com/kundajelab/deeplift	[197]	5
SpliceRover	Classification of donor and acceptor splice site	http://bioit2.irc.ugent.be/rover/splicerover/	[199]	5
RIDDLE	Imputation technique using deep learning	https://github.com/jisungk/RIDDLE	[200]	5
P-NET	Classification of prostate cancer	https://github.com/marakeby/pnet_prostate_paper	[203]	5
SHAP	An explainable machine learning tool	https://github.com/slundberg/shap	[204]	5
devCellPy	Classification of cell types across complex annotation hierarchies	https://github.com/devCellPy-Team/devCellPy	[205]	5
BCrystal	An interpretable sequence-based protein-crystallization predictor	https://github.com/raghvendra5688/BCrystal	[206]	5
MetaNet	Metastatic-risk assessment of a primary tumor	https://github.com/WangLabHKUST/METANET-analysis	[207]	5
Ocelot	Prediction of relationships across histone modifications	https://github.com/GuanLab/Ocelot	[208]	5
DeepHF	Optimization of CRISPR guide RNA design using deep learning for two high-fidelity Cas9 variants	https://github.com/izhangcd/DeepHF http://www.deephf.com/#/home	[209]	5
MTGCN	Identification of cancer-driver genes	https://github.com/weiba/MTGCN	[213]	5
GNNExplainer	An explainable graph neural-network tool	https://github.com/RexYing/gnn-model-explainer	[215]	5
SBMClone	Identification of tumor clones in sparse single-cell-mutation data	https://github.com/raphael-group/SBMClone	[221]	5
Mix-MMM	Identification of mutation signatures from sparse mutation data	https://github.com/itaysason/Mix-MMM	[222]	5
JDINAC	Identification of differential interaction patterns of network activation using high-dimensional sparse omics data	https://github.com/jijiadong/JDINAC	[223]	5
MoGP	Identification of patterns in amyotrophic lateral-sclerosis progression from sparse longitudinal data	https://github.com/fraenkel-lab/mogp	[225]	5
	Multi-cancer analysis of clonality in paired primary tumors and metastases	https://github.com/cancersysbio/pan-metastasis	[251]	5
CHESS	Spatial stochastic tumor-growth model to simulate multi-region sequencing data derived from spatial sampling of neoplasm	https://github.com/kchkhaidze/CHESS.cpp	[256]	5

## Figures and Tables

**Table 1:** Clinical applications of mutation signatures.

Category	Descriptive Mutational Process	Clinical Use
Clinically relevant DDR pathways	Homologous recombination (HR)	Biomarker for PARP-inhibitor sensitivity [64,65,66]
		Biomarker for platinum-treatment sensitivity [67]
		Biomarker for ATRi-inhibitor sensitivity [71,73,74,75]
	Mismatch repair (MMR)	Immune-checkpoint-inhibitor biomarker [77]
		Identification of Werner-helicase-sensitive patients [78,82,83]
		Potential biomarker for antitumor immune activation [84]
	Nucleotide excision repair (NER)	Biomarker for platinum-treatment sensitivity [34,85]
		Biomarker of ERCC2 deficiency [34,85]
	Proofreading errors	Biomarker of POLE deficiency [86,87]
Characterization of clinically relevant phenomena	Radiation treatment	Identification of radiation-driver tumors [53]
		Identification of genes with potential contra-indications of radiation therapy [54,88]
	Chemotherapy	Tumorigenic effects of 5-FU [88,89]
		Tumorigenic effects of platinum and capecitabine treatments
	Environmental	Screening for aristolochic-acid damage [90,91,92]
		Alcohol-consumption signatures across cancers [93,94,95,96]
	Cancer-type specific mutagenesis	Identification of different subtypes of esophageal cancer [97]
		Identification of secondary tumors of unknown origin [98]

## Data Availability

Not applicable.
